# Exploring the bioactive compounds of *Carica papaya* leaves: phytol’s role in combatting antibiotic-resistant bacteria

**DOI:** 10.3389/fcimb.2025.1564787

**Published:** 2025-07-07

**Authors:** Fahad Saad Alhodieb, Maira Farid, Maimoona Sabir, Sobia Nisa, Sumaira Sarwar, Sidra Abbas

**Affiliations:** ^1^ Department of Basic Health Sciences, College of Applied Medical Sciences, Qassim University, Buraydah, Saudi Arabia; ^2^ Department of Microbiology the University of Haripur, Haripur, KPK, Pakistan; ^3^ Islamabad Medical and Dental College, Bharakhau, Islamabad, Pakistan; ^4^ Microbiology and Biotechnology Research laboratory, Department of Biotechnology, Fatima Jinnah Women University, Rawalpindi, Pakistan

**Keywords:** antibiotic resistance of bacterial strains, inflammation, gastrointestinal infections, *Carica papaya*, antimicrobial compounds, molecular docking

## Abstract

**Introduction:**

Antibiotic resistance is a pressing global challenge, complicating the treatment of infectious diseases caused by multidrug-resistant microorganism. For centuries, medicinal plants have been a cornerstone of natural remedies, offering bioactive compounds with therapeutic potential.

**Methods:**

The study investigate the phytochemical screening and antibacterial efficay of *Carica papaya* leaf extract, focusing on its impact against three Gram-negative bacterial pathogens i.e., *Escherichia coli, Helicobacter pylori*, and *Salmonella enterica* serovar Typhi, are major contributors to gastrointestinal infections worldwide, often leading to severe inflammation and chronic health complications.

**Results:**

The phytochemical screening revealed the presence of phenols and flavonoids, which are key contributors to the extract’s biological activity. GC-MC analysis identified 27 bioactive compounds, with phytol emerging as a prominent constituent, detected at a peak retention time of 18.712 minutes. Antibacterial assays demonstrated significant efficacy, with inhibitory zones ranging from 10 to 20 mm against the tested pathogens. Molecular docking further highlighted phytol’s strong binding affinities to crucial bacterial proteins, including DNA gyrase (*E. coli*), Vacuolating cytotoxin A (*H. pylori*) from, and Dihydrofolate reductase (*Salmonella enterica* serovar Typhi). Notably, phytol exhibited the highest binding energy (-6.64 kcal/mol) with DHFR, indicating a robust interaction that underscores its potential as a targeted antibacterial agent against *Salmonella enterica* serovar Typhi.

**Discussion:**

These findings position phytol as a promising lead compound for developing novel antibacterial therapies. Its strong activity against multidrug-resistant pathogens suggests potential for further exploration, though additional research is needed to assess its role in resistance modulation or prevention.

## Graphical Abstract

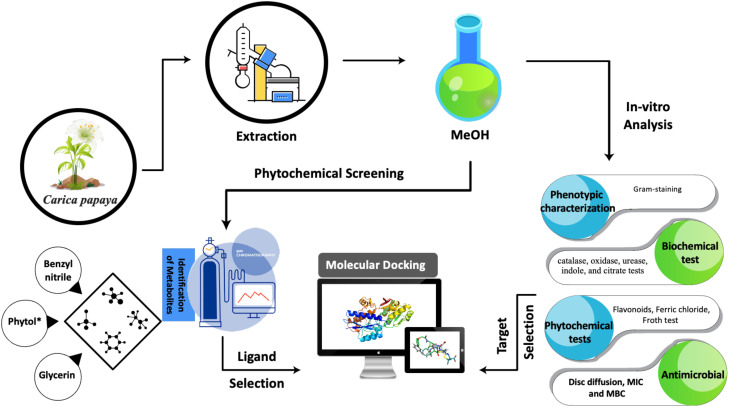

## 1 Introduction

Antimicrobial resistance (AMR) is a critical global health concern affecting several pathogens, including particularly *Helicobacter pylori (H. pylori)*, *Escherichia coli* (*E. coli*), and *Salmonella enterica* serovar Typhi (S. Typhi). In *H. pylori*, resistance to antibiotics such as metronidazole, fluoroquinolones, and clarithromycin threatens effective eradication therapy, emphasizing the need for alternative treatments and resistance monitoring (Argueta et al., 2022). Similarly*, E. coli* exhibits resistance to beta-lactams, fluoroquinolones, and carbapenems, with multidrug-resistant (MDR) and extensively drug-resistant (XDR) strains limiting treatment options for sepsis, urinary tract infections, and other infections (Abbas et al., 2023). Factors such as antibiotic overuse, poor infection prevention and control strategies contribute to this issue (Magiorakos et al., 2012). *S. enterica* serovar Typhi has also shown increasing resistance to commonly used antibiotics, including ampicillin, chloramphenicol, and co-trimoxazole (Sulfamethoxazole and trimethoprim combination), with rising resistance to third-generation cephalosporins and fluoroquinolones (Qamar et al., 2018; Chatham-Stephens, 2019). The appearance of antimicrobial resistance can be ascribed to anthropological, environmental and genetic factors. Human-related factors such as antibiotic overuse and missuse, poor infection prevention and control strategies contribute to antibiotic resistance (Magiorakos et al., 2012). The spread of AMR in S. Typhi is driven by factors like improper antibiotic use, inadequate infection control, poor sanitation and hygiene in public settings (Klemm et al., 2018). AMR is driven by a variety of genetic factors, both intrinsic and acquired, that allow bacteria to survive and thrive in the presence of antibiotics. These include mutations in genes, mobile genetic elements like plasmids, and the spread of resistance genes through horizontal gene transfer (Ahmad et al., 2024).

Plants have long been a source of medicinal agents due to their bioactive compounds, such as alkaloids, flavonoids, and terpenoids, which exhibit anti-inflammatory, antimicrobial, antioxidant, and anticancer properties (Newman and Cragg, 2016). Among these plants, *Carica papaya* is particularly notable for its rich nutritional and bioactive profile, including vitamins A and C, with trace elements such as potassium, and the enzyme papain (Santana et al., 2019). Papaya’s fruit, leaves, and seeds have been used in traditional medicine for their anti-inflammatory, antioxidant, and antimicrobial properties, with research confirming their potential in treating diseases such as cancer, diabetes, and gastrointestinal disorders (Kong et al., 2021). The seeds exhibit potent antimicrobial effects against Gram-negative bacteria (Ugbogu et al., 2023), while the leaves contain flavonoids that contribute to anti-inflammatory and antioxidant activity (Sharma et al., 2022). The fruit pulp is rich in papain and vitamin C, which are linked to gastrointestinal benefits (Kim et al., 2024). Additionally, papaya holds significant value in the food and pharmaceutical industries, making it a versatile plant for medicinal, nutritional, and industrial applications (Alara et al., 2022).

Molecular docking is a computational technique used to predict the binding affinity and orientation of small molecules within a protein’s active site, playing a crucial role in drug discovery by enabling virtual screening of large compound libraries to identify potential leads for specific protein targets (Meng et al., 2011). This method considers various factors such as hydrogen bonding, hydrophobic interactions, and electrostatic forces to predict the binding energy and conformation of the ligand-protein complex (Morris et al., 2009). Recent advances have improved its accuracy, allowing precise prediction of binding modes and affinities, and have been applied in drug design for diseases like cancer, malaria, tuberculosis, and influenza (Chen et al., 2020; Ejalonibu et al., 2021). Protein-ligand binding, an essential process in biological functions like signal transduction and enzyme inhibition, involves the recognition of molecular features such as shape, charge, and hydrophobicity, with interactions including hydrogen bonding, ionic forces, and van der Waals interactions determining the binding affinity and specificity (Chen et al., 2016). Molecular dynamics simulations and thermodynamic integration have been employed to study binding free energy and kinetics, offering insights into binding mechanisms essential for designing therapeutic agents that modulate protein function (Lazim et al., 2020).

This study investigated the antibacterial activity of *Carica papaya* leaf extract against three clinically important bacterial pathogens*, Helicobacter pylori, Escherichia coli*, and *Salmonella enteric*a serovar Typhi. It identified the bioactive compounds responsible for the antibacterial activity and elucidated the molecular interactions between these bioactive compounds and the targeted bacterial proteins using molecular docking simulations, with the ultimate goal of identifying potential lead compounds for developing novel antibacterial therapeutics.

## 2 Materials and methods

### 2.1 Inoculation of selected clinical strains

The strains of *Helicobacter pylori* (ATCC 43504) are commonly used for pathogenicity studies (Kinoshita-Daitoku et al., 2020), *Escherichia coli* (ATCC 25922) (Jafari et al., 2021; Asghar et al., 2024), and *Salmonella enterica* serovar Typhi (6539) (Asghar et al., 2024), were obtained from laboratory the department of Microbiology, The University of Haripur Khyber-Pakhtunkhwa Pakistan. *H. pylori* was cultured in Columbia blood agar supplemented with Dent media, then incubated at 37°C for one week under microaerophilic conditions (Alkharsah et al., 2022). *Salmonella enterica* serovar Typhi was cultured into the *Salmonella-Shigella* Agar and incubated for about 24 hours at 37°C (Ruiz et al., 1996). The strain of *E. coli* was cultured into the MacConkey Agar and incubated for about 24 hours at 37°C (Supriatin et al., 2021).

### 2.2 Biochemical tests

The biochemical characterization of bacterial isolates included the catalase, oxidase, urease, indole, methyl red, and citrate tests, performed following standard protocols (MacFaddin, 2000). The tests involved the addition of reagents to observe the color shift in the medium, appearance of ring and observing the bubble formation.

### 2.3 Plant materials

*Carica papaya* leaves were collected from KTS (Khalabat township) Haripur, Khyber-Pakhtunkhwa. KTS is one of the 30 union councils of Haripur District in the Khyber Pakhtunkhwa of Pakistan with coordinates: 34°13′N 73°02′ECoordinates: 34°13′N 73°02′E

The leaves were thoroughly washed three times with distilled water to remove any impurities and then dried under shade at room temperature. After drying, the leaves were ground into a fine powder using pestle and mortar. The resulting powder was stored in a glass container for further use.

### 2.4 Leaf extraction

Leaf extracts were prepared following the standard protocol described by Harborne (1998). Twenty grams of dried leaves powder were soaked in 200ml of 80% methanol for one week at room temperature, with the mixture stirred daily using a stirrer. After one week, the mixture was filtered using Whatman filter No.1 filter paper. The filtered extract was then evaporated in a hot air oven for 24 hours at 37°C. The dried leaves extract was stored in sealed bottle at 4°C for further use.

### 2.5 Phytochemical screening test

Phytochemical screening of *Carica papaya* leaf extract was conducted to identify secondary metabolites (Sofowora, 1993).

### 2.6 Gas chromatography-mass spectrometry analysis

The chemical composition of plant extract was assessed using GC-MS on a Perkin Elmer Clarus 600 GC with 600c MS. Two fused silica capillary columns Elite 5-MS (30 m) were used. The oven temperature was initially set to 50°C for 2 minutes and then programmed to 300°C at a rate of 5°C/min. The injector temperature was set between 220 and 250°C. A 1 μl diluted sample(in chloroform) was injected with a 100:1 split ratio. Helium served as the carrier gas (2 mL/min), and analysis utilized the NIST (National Institute of Standards and Technology) library database (Nisa et al., 2020).

### 2.7 Antimicrobial activity

The antimicrobial activity was assessed using a modified agar well diffusion technique adapted from the standardized disk diffusion method originally developed by Heatley (1944) and subsequently refined for clinical applications (Balouiri et al., 2016). Mueller-Hinton Agar (MHA) plates were prepared according to CLSI guidelines and sterilized by autoclaving at 121°C for 15 minutes. Following solidification, the plates were inoculated with standardized bacterial suspensions (1.5 × 10^8^ CFU/mL) of the test organisms including *H. pylori*, *S. enterica serovar* Typhi, and *E. coli*, using sterile swabs to create uniform bacterial lawns.

Four equidistant wells (6 mm diameter) were aseptically punched into each agar plate using a sterile cork borer (Nurkhaliza et al., 2024). To prevent lateral diffusion of test compounds while maintaining well integrity, 20 μL of molten MHA was carefully layered at the base of each well and allowed to solidify. The wells were then loaded with three concentrations of the methanolic leaf extract (5 mg/mL, 50 mg/mL, and 100 mg/mL, 100 μL per well) and a negative control (100 μL DMSO) in the fourth well. Antibiotics (amoxicillin, ceftriaxone, and gentamicin) were used as positive control against control for *H. pylori, Salmonella enterica* serovar Typhi and *E. coli* respectively. All plates were incubated at 37°C for 24 hours (microaerophilic conditions for *H. pylori*), after which the diameters of inhibition zones were measured to the nearest millimeter using digital calipers. Each assay was performed in triplicate to ensure reproducibility.

### 2.8 Determination of MIC and MBC

The minimum inhibitory concentration (MIC) of *Carica papaya* leaves extract was determined using microdilution method in a 96-well flat-bottom plate (Bauer et al, 1966; Kowalska-Krochmal and Dudek-Wicher, 2021). Wells from A1 to C1 served as negative controls containing 100 µl of nutrient broth. In wells A3 to C12, 100 µl of nutrient broth was dispensed, and serial dilutions of the plant extract were prepared across these wells. A volume of 100 µl of each of the three test pathogens was inoculated into wells A2 to C12. The plate was incubated at 37°C for 24 hours, after which the optical density of each well was measured using spectrophotometry at 520nm to calculate the percentage inhibition.

The minimum bactericidal concentration (MBC) was determined by plating of 20 µl of each dilution from the microdilution assay onto MHA (Mogana et al., 2020). The plates were incubated at 37°C for 24 hours. After incubation, the presence or absence of the bacterial growth was recorded by visual inspection, and zones of inhibition were measured to confirm the bactericidal effect of the extract.

### 2.9 Selection of ligand and protein

Phytol was selected as the ligand based on its high abundance in the GC-MS analysis of *Carica papaya* leaf extract. Among the 27 identified compounds, Phytol exhibited the highest peak, indicating its dominance in the extract.

For the target proteins, one representative protein was selected from each of the selected bacterial pathogens. VacA protein from *H. pylori*, Dihydrofolate Reductase (DHFR) from *Salmonella enterica* serovar Typhi, and DNA gyrase from *E. coli* were chosen due to their critical roles in bacterial virulence or survival.

### 2.10 Protein preparation

The protein structures were carefully prepared for docking simulations to ensure accurate analysis with bioactive compounds (Sastry et al., 2013). Relevant protein sequences of the selected pathogens were identified through the National Centre for Biotechnology Information (NCBI) database. These sequences were then used to retrieve the corresponding two and three-dimensional structures from the Protein Data Bank (PDB), providing reliable templates for the docking studies. Protein preparation involved the removal of non-essential chains and water molecules, followed by the addition of polar hydrogens and the application of Kolman and Gasteiger charges. The modifications were performed using AutoDock tools and Chimera to ensure the proteins were ready for molecular docking analysis (Abbas et al., 2024). This thorough preparation process ensured the structural integrity and compatibility of the proteins for downstream molecular docking simulations, facilitating precise analysis.

### 2.11 Ligand preparation

To prepare the ligands, PubChem was accessed to retrieve the accurate chemical structures and details of the bioactive compound derived *Carica papaya* leaves extract. The ligand structure was then converted into suitable format using OpenBabel, ensuring compatibility with the docking software (O’Boyle et al., 2011). Subsequently, Chimera and AutoDock tools were employed to refine both the protein and ligand structures, addressing any structural issues and optimizing the molecular orientations (Butt et al., 2020). This thorough preparation process ensured generation of high-quality inputs for docking simulations, enabling precise analysis of the interactions between the bioactive compounds and bacterial targets.

### 2.12 Molecular docking

Molecular docking was utilized as a pivotal in-silico technique to investigate the potential mechanisms of action of the bioactive compounds in *Carica papaya* leaves (Arwansyah et al., 2025). This computational approach simulated the interaction between the identified compounds and key bacterial proteins, providing valuable insights into their binding affinity and specificity. The docking simulations identified potential binding sites and interaction modes, thereby enhancing our understanding of the extract’s efficacy and guiding further experimental validation. Version 4.2.6 of AutoDock was employed to prepare the grid boxes and map files for docking analysis (Abbas et al., 2024). Grid parameter files (GPF) were generated with X, Y, and Z coordinates defining a grid size of 60 x 60 x 60, ensuring sufficient search space for ligand flexibility and rotation. Additional atomic map types, including hydrogen (H), chlorine (Cl), bromine (Br), sulphur (S), phosphorus (P), and fluorine (F), were included to construct the grid box surrounding the active site of the target protein. MGL Tools version 1.5.7 was used to generate the necessary atomic map files by processing the GPF files. Subsequently, grid log files (GLG) containing all atomic maps were created via AUTOGRID. These files served as input for docking calculations, enabling a detailed evaluation of ligand-receptor interactions. The resulting docking conformations were analyzed using Discovery Studio Visualizer, Version 3.1, where 10 docking conformations of the phytol-protein complexes were thoroughly examined to evaluate the potential interactions between the ligand and the bacterial proteins. This systemic approach provided a robust framework for understanding the bioactive potential of the compounds and offered critical insights for subsequent experimental research.

### 2.13 Pharmacokinetic parameters.

The phytol compound was assessed drug-likeness and pharmacokinetic properties using advanced computational tools. SwissADME (http://www.swissadme.ch) (Daina et al., 2017), was utilized to predict properties such as synthetic accessibility, absorption, and metabolism. Additionally, pkCSM was employed to evaluate key pharmacokinetic parameters, including intestinal absorption, volume of distribution (VDss), CYP metabolism, total clearance (Blundell and Ascher, 2015). Toxicity predictions were performed using ProTox-II (Banerjee et al., 2018), and StopTox (Borba et al., 2022). ProTox-II provided insights into endpoints like carcinogenicity, mutagenicity, acute toxicity, hepatotoxicity, cytotoxicity, immunotoxicity, adverse outcomes (Tox21) pathways, and toxicity targets. Its methodology integrated fragment-based propensities, molecular similarity, frequently observed features, and machine learning algorithms. StopTox applied advanced quantitative structure-activity relationship (QSAR) models to predict toxicity endpoints. StopTox specifically addressed concerns such as inhalation and oral toxicity, skin irritation, and sensitization by using extensive publicly available datasets on toxicity (Borba et al., 2022).

These computational evaluations provided a comprehensive understanding of the pharmacokinetic behavior, drug-likeness, and safety profiles of phytol, supporting its potential as a therapeutic candidate.

## 3 Results and discussion

### 3.1 Phenotypic features of selected strains

**Figure 1:**
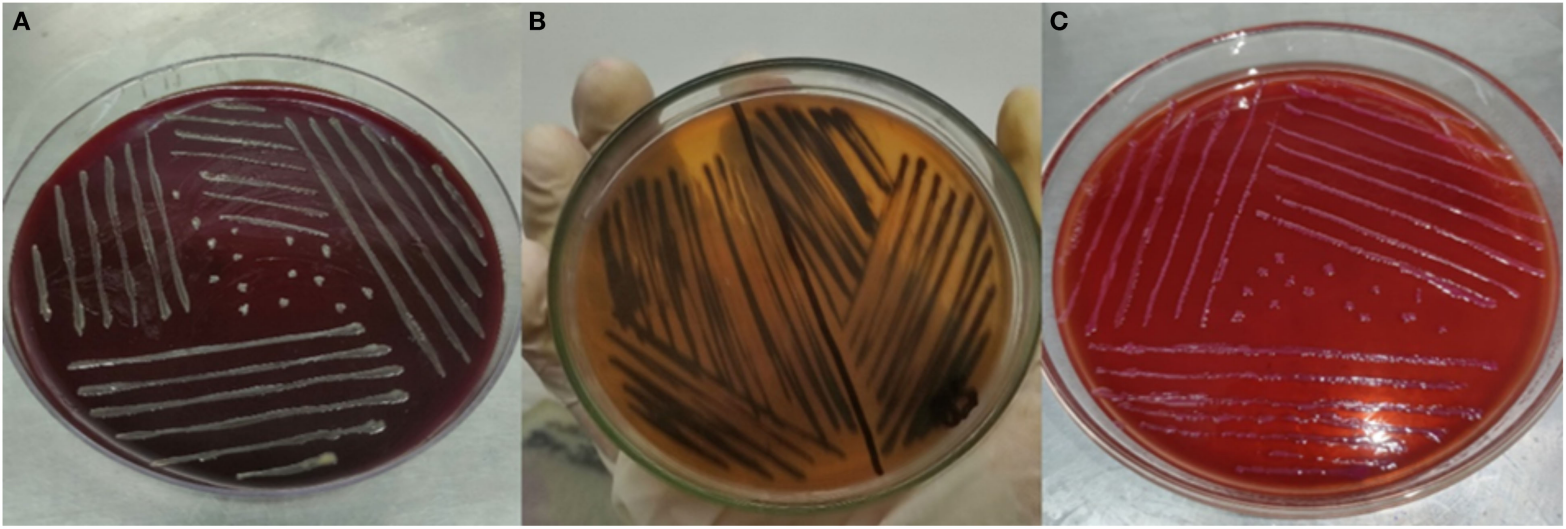
**(A)** *H*. *pylori* on Columbia blood agar. **(B)** *Salmonella enterica* serovar Typhi on *Salmonella-Shigella* agar. **(C)** *E*. *coli* on MacConkey Agar.

Distinct colonies of each bacterial strain were observed on specific culture media. *H. pylori* formed white to greyish colonies on Columbia blood agar, *Salmonella enterica* serovar Typhi displayed black colonies on *Salmonella-Shigella* agar, and *E. coli* produced pink colonies on MacConkey Agar, as illustrated in **Figure 1**. All pathogens reacted with pink color after Gram-staining confirming them as Gram-negative bacteria (Tripathi and Sapra, 2020). *H. pylori* appeared as small, curved or spiral-shaped cells (Krzyżek and Gościniak, 2018), *Salmonella enterica* serovar Typhi as single rods or occasionally in short chains (Arunava Das et al., 2012), and *E. coli* as single rods, with occasional pairs or short chains (Islam et al., 2016).

### 3.2 Biochemical testing

The biochemical test results reveal distinct metabolic characteristics of *H. pylori*, S. Typhi, and *E. coli* showcasing their adaptations to specific ecological niches and metabolic pathways (**Table 1**). The catalase positive results for all three bacteria confirm their ability to degrade hydrogen peroxide, a mechanism essential for combating oxidative stress, particularly in environments exposed to reactive oxygen species. *H. pylori*, for instance, encounters high levels of oxidative stress in the stomach’s acidic environment, and catalase activity is critical for its survival (Benoit and Maier, 2016). Similarly, S. Typhi, and *E. coli*, as gut-associated organisms, require catalase for defense against oxidative bursts from host immune response (**Supplementary Figure S1**) (Chanin et al., 2020; Hahn et al., 2021).

**Table 1:** Biochemical test results.

Bacteria	Catalase	Oxidase	Urease	Indole	MR	Citrate
*H. pylori*	**+**	**+**	**+**	**-**	**-**	**-**
*S.* Typhi	**+**	**-**	**-**	**-**	**+**	**-**
*E. coli*	**+**	**-**	**-**	**+**	**+**	**-**

*E. coli* is oxidase-negative, characteristic of the *Enterobacteriaceae* family, which relies on facultative anaerobic and can switch between aerobic respiration and fermentation depending on oxygen availability (Zeng et al., 2017). Urease positive activity of *H. pylori* shows its ability to survive in the acidic gastric environment by neutralizing acid with ammonia, which it produces from urea (Berger, 2000; Kuo et al., 2015). This adaptation is crucial for colonization and pathogenesis in stomach, a unique feature not shared by *S.* Typhi, and *E. coli* (Ansari and Yamaoka, 2017; Cheok et al., 2021). The inability of all three bacteria to utilize citrate as the sole carbon source is notable. While some *E. coli* strains exhibit citrate utilization under aerobic conditions, this variability shows the influence of specific environmental factors on metabolic capabilities. Among the tested strains only *E. coli* showed the ability to produced indole which was confirmed by the formation of purple ring (**Supplementary Figure S1C**).

### 3.3 Phytochemical profile

Phytochemical profile of *Carica papaya* leaves revealed presence of different bioactive compounds, including phenols and flavonoids, while saponins were absent (**Table 2**). Phenols are known for their antimicrobial, antioxidant, and anti-inflammatory properties, which could contribute to the observed biological activity of the extract (Kauffmann and Castro, 2023). Their presence is indicated by a characteristic brownish-black color reaction with ferric chloride (**Supplementary Figure S2**). Flavonoids, a class of secondary metabolites, possess well-documented biological activities, including, antimicrobial, antioxidant, and anti-inflammatory effects (Zandavar and Babazad, 2023). The color change observed upon the addition of NaOH solution signifies their presence. Studies have indicated that flavonoids can disrupt bacterial cell walls, inhibit enzymes, or interfere with quorum-sensing mechanisms in bacteria (Nguyen et al., 2024). These findings align with previous studies, such as Prasad et al. (2021), which reported similar results, highlighting the therapeutic potential of the extract.

**Table 2:** Phytochemical profile of *Carica papaya* leaves extract.

Phytochemical Tests	Results
Test for phenols (Ferric chloride test)	+ve
Test for flavonoids	+ve
Test for saponins (Froth test)	-ve

Phytochemical profile of *Carica papaya* leaves extract showed that flavonoids and phenols are present in the extract, while saponins are absent, as shown in **Table 2**.

**Figure 2:**
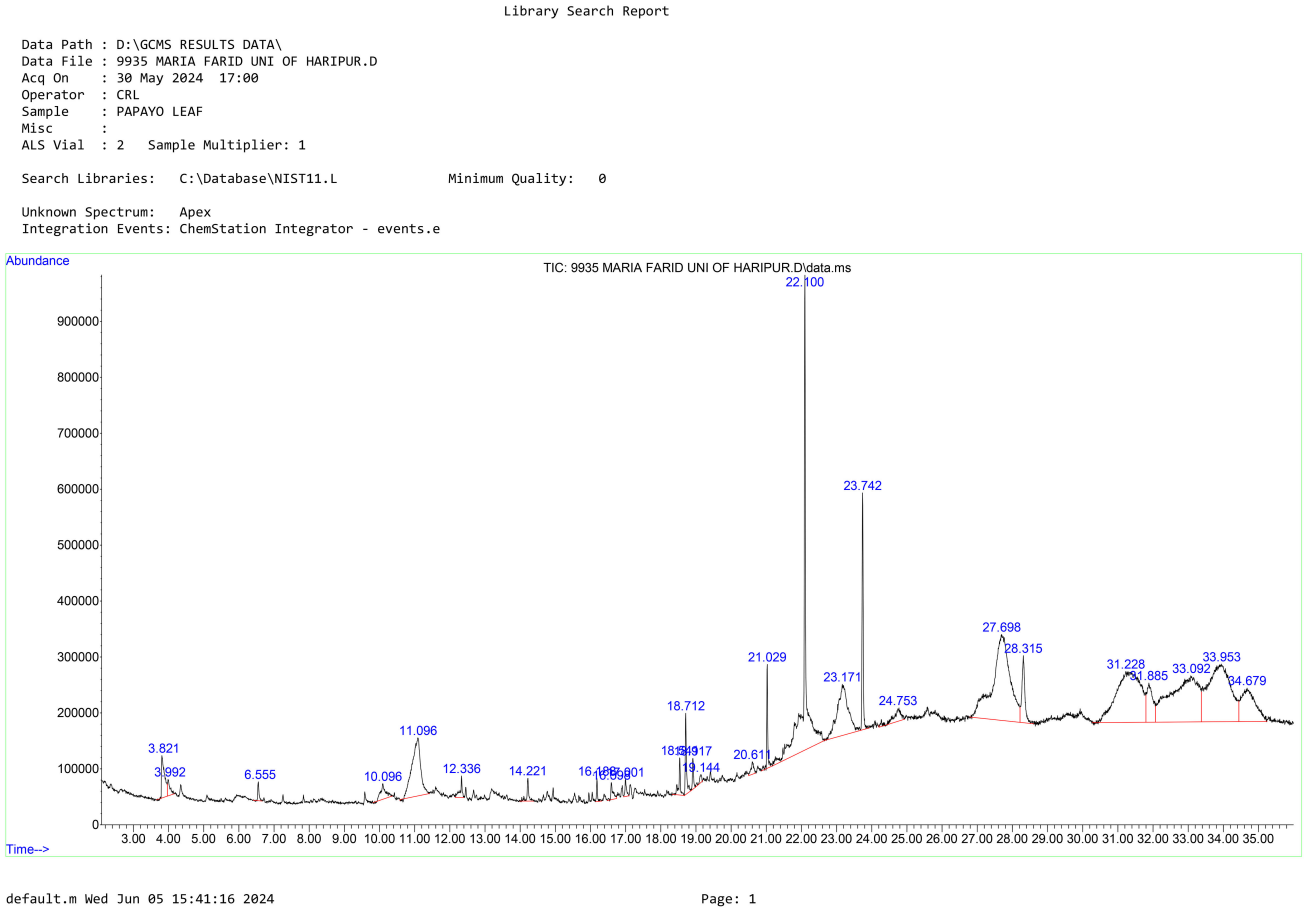
The chromatogram shows peak values of the compounds identified from *Carica papaya* leaves extract. Peak 22.100 represents phytol which is a compound commonly found in papaya leaves.

The GC-MS analysis of *Carica papaya* leaves extract, depicted in the chromatogram (**Figure 2**), reveals distinct peaks at specific retention times, highlighting the diverse phytochemical composition of the extract, with phytol dominating as the highest peak at a retention time of 18.712 minutes (**Figure 2**). The analysis identified Phytol, Benzene-(isothiocyanatomethyl), and 9-Octadecenoic acid, methyl ester (E) as the principal bioactive compounds, all known for their antibacterial properties (Al-Seadi et al., 2021). Phytol which was prominent in the identified compounds in the extract is known to be essential in plant defense processes and therefore is a good candidate for possible therapeutic activity (Ghaneian et al., 2015).

Based on these findings, Phytol was selected for further testing to evaluate its antibacterial activity against the three selected bacterial strains. Phytol is a potent bioactive compound known for its antioxidant, antimicrobial, and anti-inflammatory properties. Phytol has also been linked to potential anticancer activity, further emphasizing its pharmacological significance. Other peaks observed in the chromatogram, corresponding to different retention times, likely represent additional phytochemicals such as flavonoids, phenols, saponins, and alkaloids (**Table 3**). These secondary metabolites are known to contribute *Carica papaya’*s therapeutic efficacy. For instance, phenols and flavonoids possess string antioxidant properties, which protect against oxidative stress and have been widely documented for their role in disease prevention (Babalola et al., 2024). Moreover, studies by Achukwu et al. (2024) have shown that *Carica papaya* leaves are rich in phytochemicals that support antimicrobial activity against a range of pathogenic bacteria and fungi. These compounds likely work synergistically, enhancing the plant’s medicinal potential.

**Table 3:** Compounds identified through GC-MS.

Compounds	Mol weight	References
Glycerin	92g/mol	C3H8O3
Benzyl nitrile	117g/mol	C8H7N
4-Butoxy-1-butanol	146g/mol	C8H18O2
1,2,4-Thiadiazol-5-amine, 3-(phenylmethyl)-	191g/mol	C9H9N3S
Phytol*	296g/mol	C20H40O
9-Octadecenoic acid, methyl ester, (E)-	296g/mol	C19H36O2

*Highest peak value of 22.100 after GCMS analysis.

### 3.4 Antibacterial activity

The antibiotics used in the study were selected based on their common use in treating selected pathogens in clinical settings of Pakistan. Amoxicillin is a widely prescribed antibiotic for treating *H. pylori*, particularly in combination therapies for eradicating *H. pylori* in peptic ulcers and gastritis (Roberts et al., 2022; Vu et al., 2022). Similarly, ceftriaxone, a third-generation cephalosporin is highly effective against *Salmonella enterica* serovar Typhi (Argimón et al., 2022). Gentamycin, an aminoglycoside, is effective against a wide range of Gram-negative, including *E. coli* (Karunarathna et al., 2024). The selection of these three antibiotics as positive control was based on their susceptibility against the selected pathogens and their use in clinical practice, allowing for a meaningful comparison with the antimicrobial efficacy of *Carica papaya* leaves extract. Amoxicillin was very effective against *H. pylori* as a zone of inhibition of 18 mm was observed. Ceftriaxone and gentamycin were moderately effective against S. Typhi 14 mm, and 19 mm for *E. coli* respectively. These findings align with research demonstrating the potential of *C. papaya* against *E. coli* (Rubaka et al., 2014). This suggest that *E. coli* might have inherent resistance to gentamycin at low concentrations but could be overcome at higher doses (Gauba and Rahman, 2023). The efficacy of *Carica papaya* leaves extract has shown great efficacy against *H. pylori, Salmonella enterica* serovar Typhi and *E. coli.* The leaf extract exhibited highest activity against *H. pylori*, with a 20 mm zone of inhibition at 5 mg/ml, which aligns with previous studies highlighting the effectiveness of natural plant extracts against *H. pylori* due to their bioactive components. For example, Prasad et al. (2021) reported similar findings where papaya leaf extract exhibited potent inhibitory effects against *H. pylori*, underscoring the plant’s traditional use in treating gastrointestinal infections. This consistency suggests that *Carica papaya* could be an effective natural alternative for treating *H. pylori-*related conditions, such as peptic ulcers and gastritis.

However, as the concentration increased, the zone decreased to 13 mm at 100 mg/ml. Similar trends were observed for *S. enterica* and *E. coli*, where the zone consistently decreased with increasing extract concentration. Notably, *E. coli* demonstrated the least susceptibility overall, with the inhibition dropping from 12 mm at 5 mg/ml to 5 mm at 100 mg/ml.

These findings are consistent with previous studies that report antibacterial activity of *Carica papaya* leaves extract, attributed to phytochemical like alkaloids, flavonoids, and tannins, which may disrupt bacterial cell walls or interfere with metabolic pathways (Lee et al., 2016; Udinyiwe and Omoregie, 2024). However, the observed decrease in efficacy with higher concentrations contrasts with some studies, which suggest a dose-dependent increase in activity, indicating possible differences in extract composition, bacterial susceptibility, or assay conditions. The phenomenon observed, where higher concentrations reduce effectiveness, might be attributed to the “paradoxical effect”, where bioactive compounds may interact more efficiently with bacterial cells at lower concentrations, but at higher levels, they could accumulate and hinder diffusion, reducing efficacy. Additionally, plant extracts may stress the bacteria, triggering defense mechanisms like efflux pumps that expels toxins from the cell, thus reducing the extract’s effectiveness, especially in Gram-negative bacteria, which are known for their robust defense mechanisms (Askarinia et al., 2019; Jubair et al., 2021). Plant extracts often contain large, hydrophobic or poorly water-soluble molecules such as fatty acids or terpenoids (e.g., phytol), which may not effectively diffuse through the agar matrix. This leads to smaller or absent zones of inhibition despite significant antibacterial activity observed in broth dilution assays (CLSI, 2012; Rios et al., 1988). At elevated concentrations, certain phytochemicals present in Carica papaya leaf extract may aggregate, reducing their bioavailability and, consequently, their antimicrobial activity. This aggregation can hinder the diffusion of active compounds into the agar medium, leading to smaller zones of inhibition. Some compounds may exhibit potent activity in liquid broth but may not diffuse well in agar, resulting in underestimation of their activity by zone of inhibition alone (Balouiri et al., 2016).

The antimicrobial activity of *Carica papaya* leaves extract against all three pathogens is shown in **Table 4**.

**Table 4:** Antibacterial activity of *Carica papaya* through well diffusion method.

Bacteria	Drug	Zone of Inhibition (mm)	Control (DMSO)	Extract concentration (mg/ml)	Zone of Inhibition (mm)
*H. pylori*	Amoxicillin	18	0	5	20
				50	16
				100	13
*S.* Typhi	Ceftriaxone	14	0	5	13
				50	12
				100	11
*E. coli*	Gentamycin	19	0	5	12
				50	10
				100	5

### 3.5 Antimicrobial activity of *Carica papaya* leaves extract: MIC and MBC evaluation

**Figure 3:**
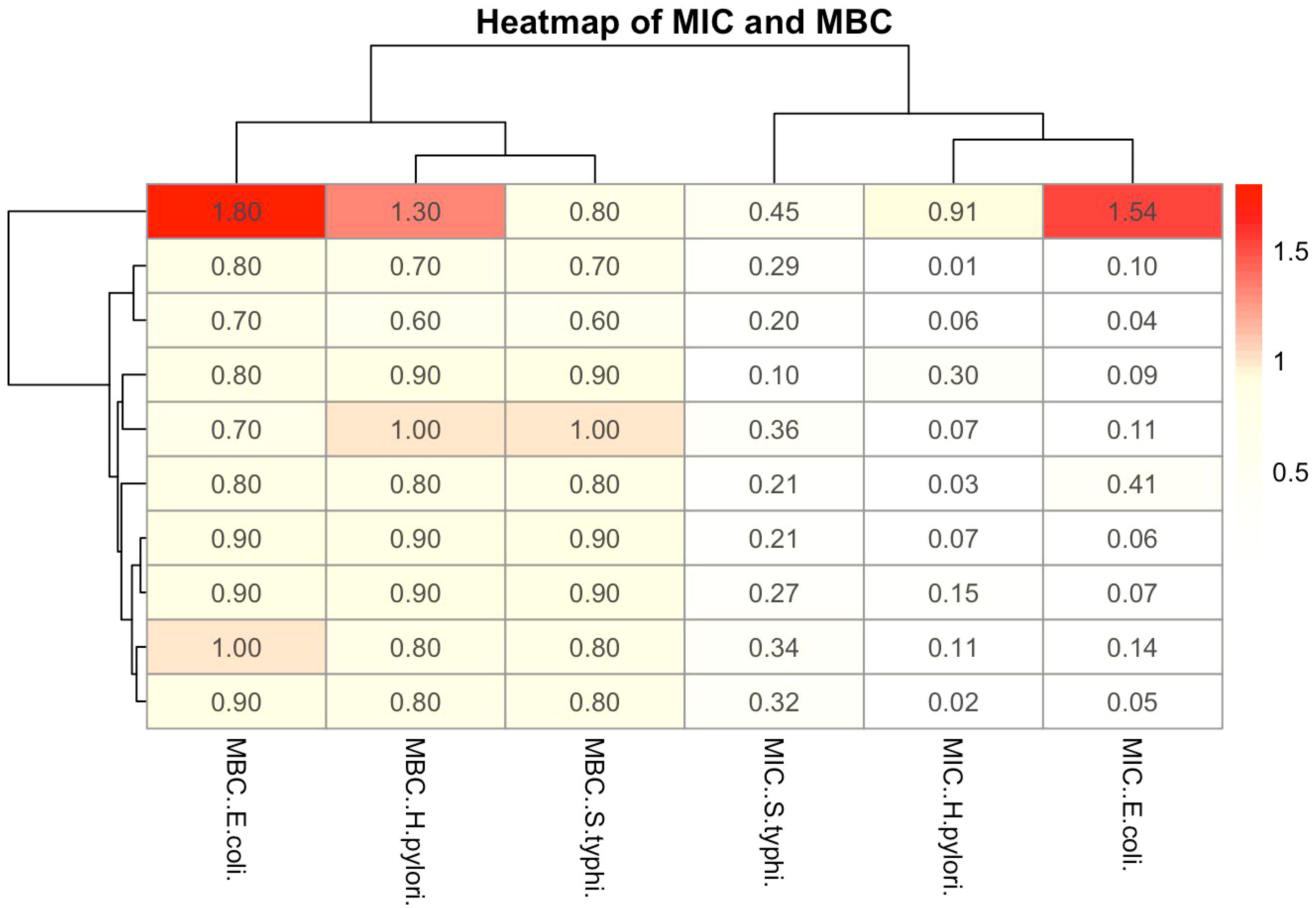
The heatmap illustrates the MIC and MBC of *Carica papaya* leaves extract against the selected pathogens. The color gradient represents the concentration values, with red indicating higher values and blue indicating lower values.

Determination of MIC and MBC of *H. pylori*, *S.* Typhi, and *E. coli* in **Figure 3** respectively. *H. pylori* show the highest MIC and MBC values (0.4-0.983), suggesting it is the least sensitive to the extract, while *E. coli* exhibits the lowest values (0.282-1), indicating higher susceptibility, S. Typhi displayed intermediate sensitivity with MIC ranging from 0.45-0.75 and MBC from 0.5-0.9. The clustering shows that MIC and MBC patterns for *H. pylori* are more similar, while S. Typhi and *E. coli* exhibit varied responses. Overall, the results suggest that *Carica papaya* leaves extract has differential antimicrobial activity.

### 3.6 Efficacy assessment of *Carica papaya* extract

**Figure 4:**
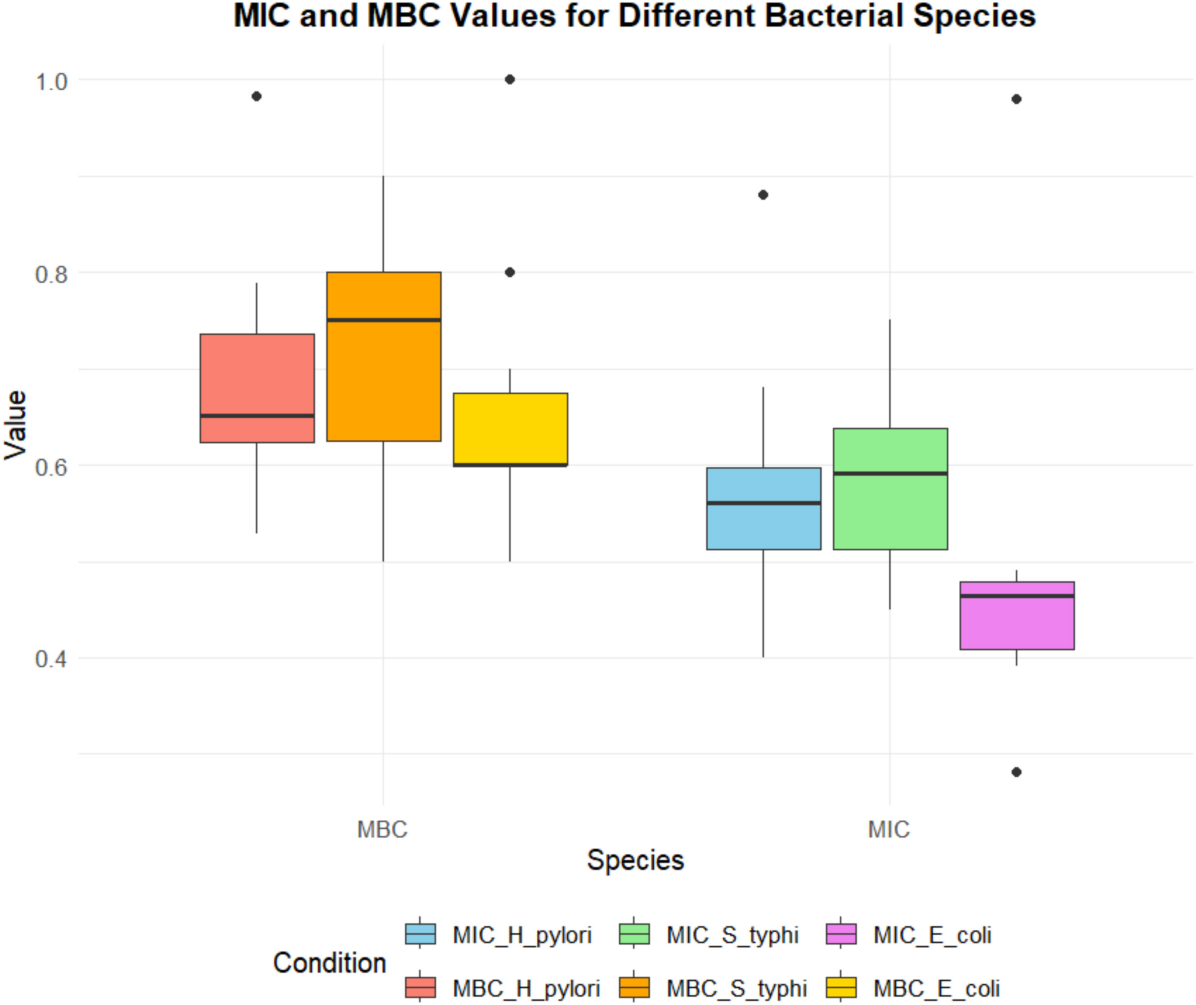
Comparison of MIC (minimum inhibitory concentration) and MBC (minimum bactericidal concentration) values of phytol for different bacterial species, including *H. pylori*, S. typhi, and *E coli.* The MIC values are represented in shades of blue, green, and purple, while the MBC values are depicted in shades of red, orange, and yellow. The plot highlights variability across species and conditions, with outliers indicating potential deviations in susceptibility or bactericidal response.

The variability in the MIC and MBC values of the leaf extract against the tested strains is illustrated in **Figure 4**. The MIC and MBC values vary significantly between bacterial strains, with S. Typhi exhibiting the lowest MIC values, suggesting a higher sensitivity to the extract. Conversely, *H. pylori* and *E. coli* exhibit slightly higher MIC and MBC values, indicating relatively reduced susceptibility. Notably outliers reflect the variability in response among the bacterial isolates, which may be attributed to intrinsic resistance mechanisms.

Yahaya et al. (2017) reported in study that the ethanolic leaf extract of *Carica papaya* exhibited increasing zones of inhibition against *E. coli*, Shigella spp., *P. aeruginosa*, and S. Typhi with increasing concentrations up to 200 mg/ml. However, beyond this concentration, a plateau or reduction in activity was noted, suggesting a potential paradoxical effect at higher concentrations. Romasi et al. (2011) research reported that the antibacterial activity of *Carica papaya* leaf extracts was influenced by p^H^, with optimal activity observed at acidic p^H^ levels. This indicates that factors such as p^H^ can significantly affect the efficacy of the extract. Evaluation of Antimicrobial Susceptibility of *Salmonella* Isolated from Household Cockroaches Using *Carica Papaya* Leaf Extract: The study found that ethanol extracts inhibited Salmonella at lower concentrations (12.5 g and 25 g) but lost effectiveness at higher concentrations (37.5 g and 50 g), indicating ethanol enhances *Carica papaya’s* antimicrobial activity at specific levels (Abutu and Gbaraba, 2021).

### 3.7 Protein preparation

**Figure 5:**
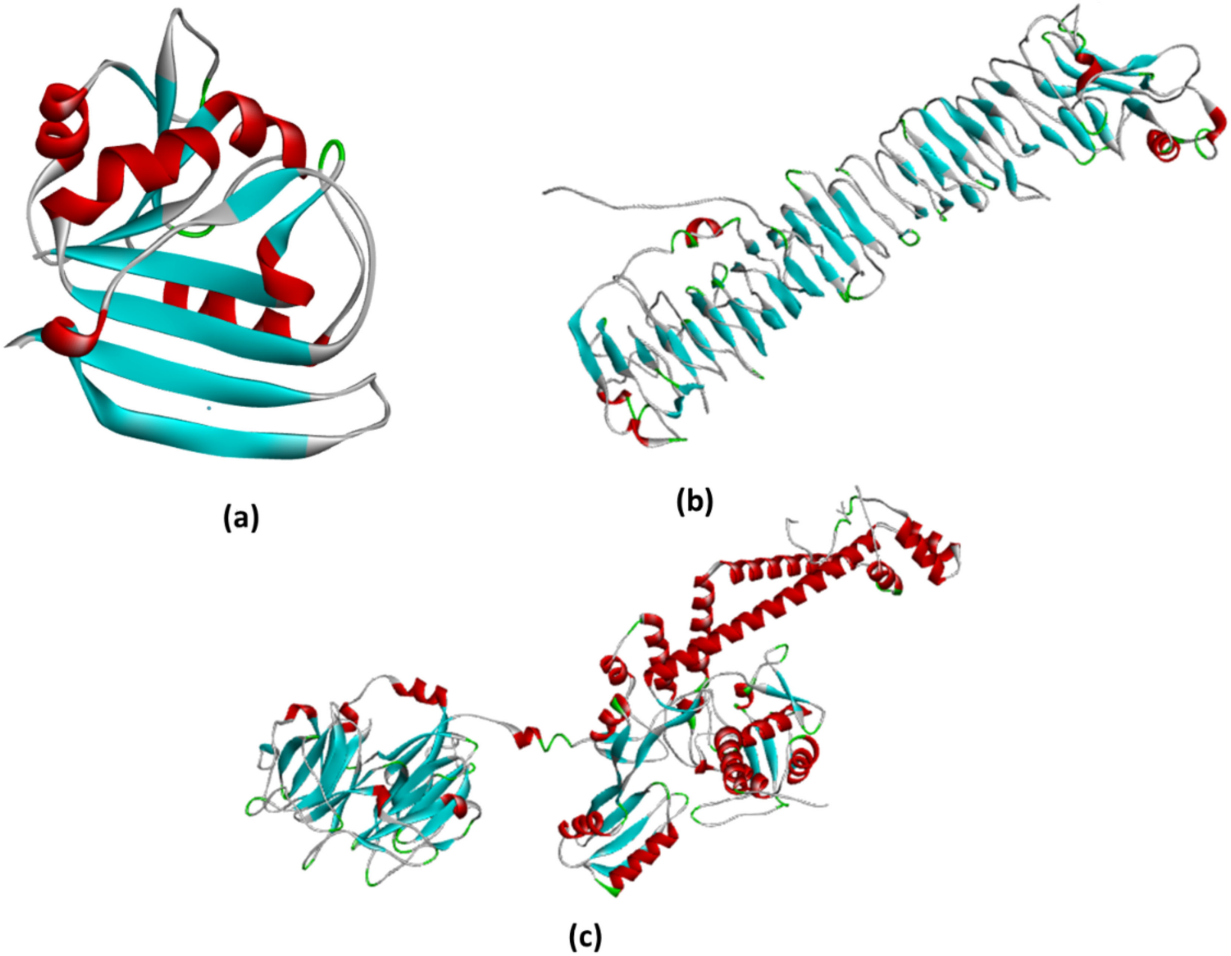
**(a)** The structure represents the prepared DHFR protein ready to bind with the ligand. All the water molecules were removed, while polar hydrogens and charges were added by using AutoDock tools; **(b)** The prepared chain from VacA ready to bind with the ligand. All chains except chain A were removed, water molecules removed, while polar hydrogens and charges were added by using AutoDock tools; **(c)** The DNA gyrase protein was prepared for docking by removing all water molecules and chains, except for chain **(a)** additionally, polar hydrogens were added, and charges were assigned using AutoDock tools to optimize the structure for docking analysis.

The 3D structure of Phytol downloaded from PubChem (https://pubchem.ncbi.nlm.nih.gov/) is shown in **Figure 5**, and the three proteins were prepared by using Auto Dock tools for docking.

### 3.8 Molecular docking

Protein-ligand interactions are influenced by various bonded and non-bonded interactions, including hydrogen bonds, electrostatic interactions, and van der Waals forces, all of which contribute to a stable protein-ligand complex. To analyze and compare the binding patterns of docked ligand within target proteins, the ligands with the highest docking scores were extracted and graphically visualized.

**Figure 6:**
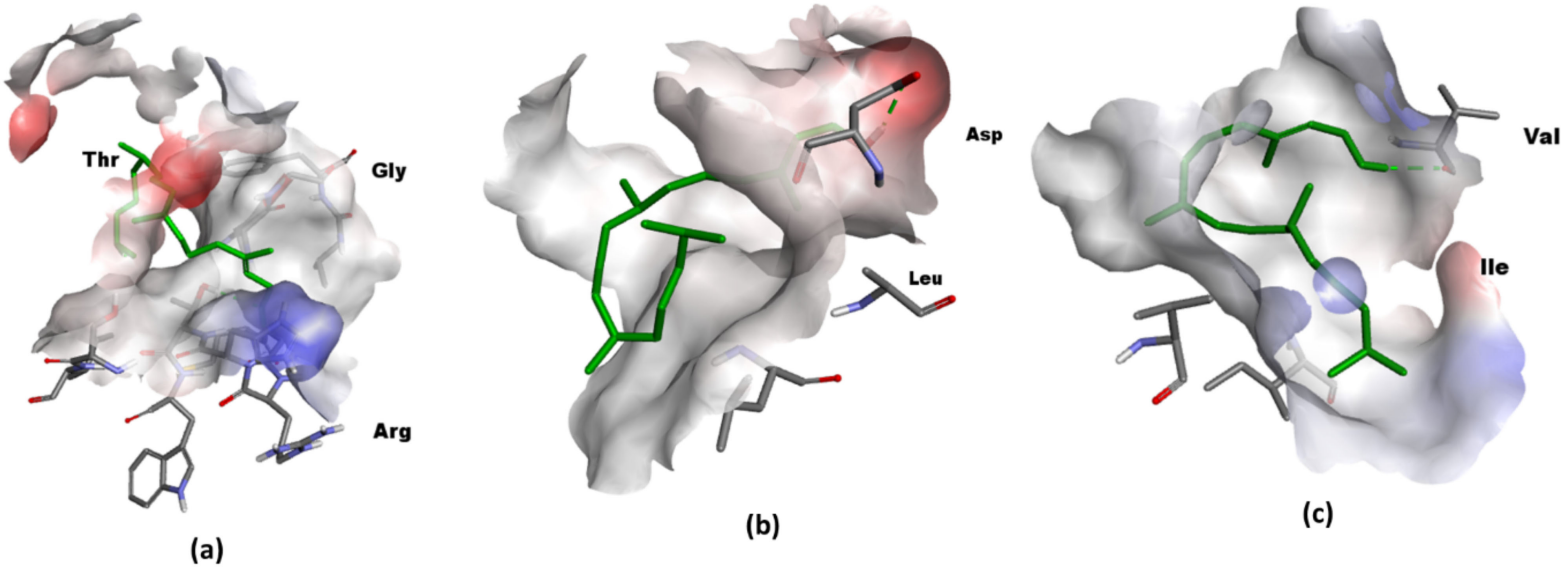
3D visualization in Discovery Studio **(a)** Phytol targeting DHFR. The prepared structure of DHFR shows Phytol binding to the protein, with two key interactions identified between the ligand and the residues GLY-96 and THR-46, at a distance of 3.1. (PDB ID: 1rx2); **(b)** 3D visualization Phytol targeting VacA. The interaction between the protein and ligand highlights ASP-346 as a interacting residue, with a measured distance 3.5 Å (PDB ID: 6nyl) **(c)** DNA gyrase with the interacting residue VAL-685 ((PDB ID: 6rkw).

**Figure 6** illustrates the optimally docked complexes of target proteins from tested bacterial strains with the phytol. The interaction of phytol with VacA identified ASP-346 as a key interacting residue and with a binding distance of 3.5 Å. For DHFR, two interactions were observed involving the residues GLY-96 and THR-46, with a binding distance of 3.1 Å. Similarly, the interaction of Phytol against DNA gyrase highlighted VAL-685 as the interacting residues.

Binding energy analyses revealed that phytol exhibited binding energies of -2.89 kcal/mol against VacA, -6.64 kcal/mol against DHFR, and -2.24 kcal/mol against DNA gyrase, as summarized in **Table 5**. These results underscore phytol’s antibacterial potential, particularly its strong binding affinity to dihydrofolate reductase (DHFR) from *Salmonella enterica* serovar Typhi (-6.64 kcal/mol). This finding aligns with Asad et al. (2021), who reported phytol’s inhibitory effect on DHFR in similar bacterial strains. In contrast, Phytol’s lower binding energies against VacA from *H. pylori* (-2.89 kcal/mol) and DNA Gyrase (GyrA) from *E. coli* (-2.24 kcal/mol) suggest that its efficacy may be more pathogen-specific. This observation is consistent with the study by Sharma et al. (2022), who reported variable binding affinities of phytol across different bacterial targets, indicating potential limitations in its broad-spectrum effectiveness.

**Table 5:** Binding energy and residue interactions of tested proteins in against phytol.

Proteins	Binding energy	H bonds	Interacting residues
VacA	-2.89	1	ASP-346
**DHFR**	**-6.64**	**2**	GLY-96 and THR-46
DNA gyrase	-2.24	1	VAL-685

Bold values means strong binding energy then others.

### 3.9 Pharmacokinetic parameters

Phytol’s pharmacokinetics properties, evaluated through in-silico ADME tools, pkCSM, Stoptox, Pro-Tox II, underscore its potential as a drug candidate. With a molecular weight of 296.53, it falls within the range suitable for drug-likeness and its polar surface area of 20.23 Å^2^ supports good membrane permeability and absorption (Almeida-Bezerra et al., 2024). Phytol’s high lipophilicity (Log *P*_o/w_ 6.25) enhances its ability to penetrate lipid membrane, while a single Lipinski rule violation suggests acceptable drug-likeness (**Table 6**).

**Table 6:** Pharmacokinetic properties of phytol.

Pharmacokinetic properties	
Molecular weight	296.53
Molar refractivity	98.94
Polar surface area (Å^2^)	20.23 Å^2^
Consensus Log *P*_o/w_	6.25
Water solubility (mg/mL)	-5.98
LogK*_p_* (skin permeation) (cm/s)	-2.29 cm/s
Lipinski violations	1
Bioavailability score	0.55

**Figure 7:**
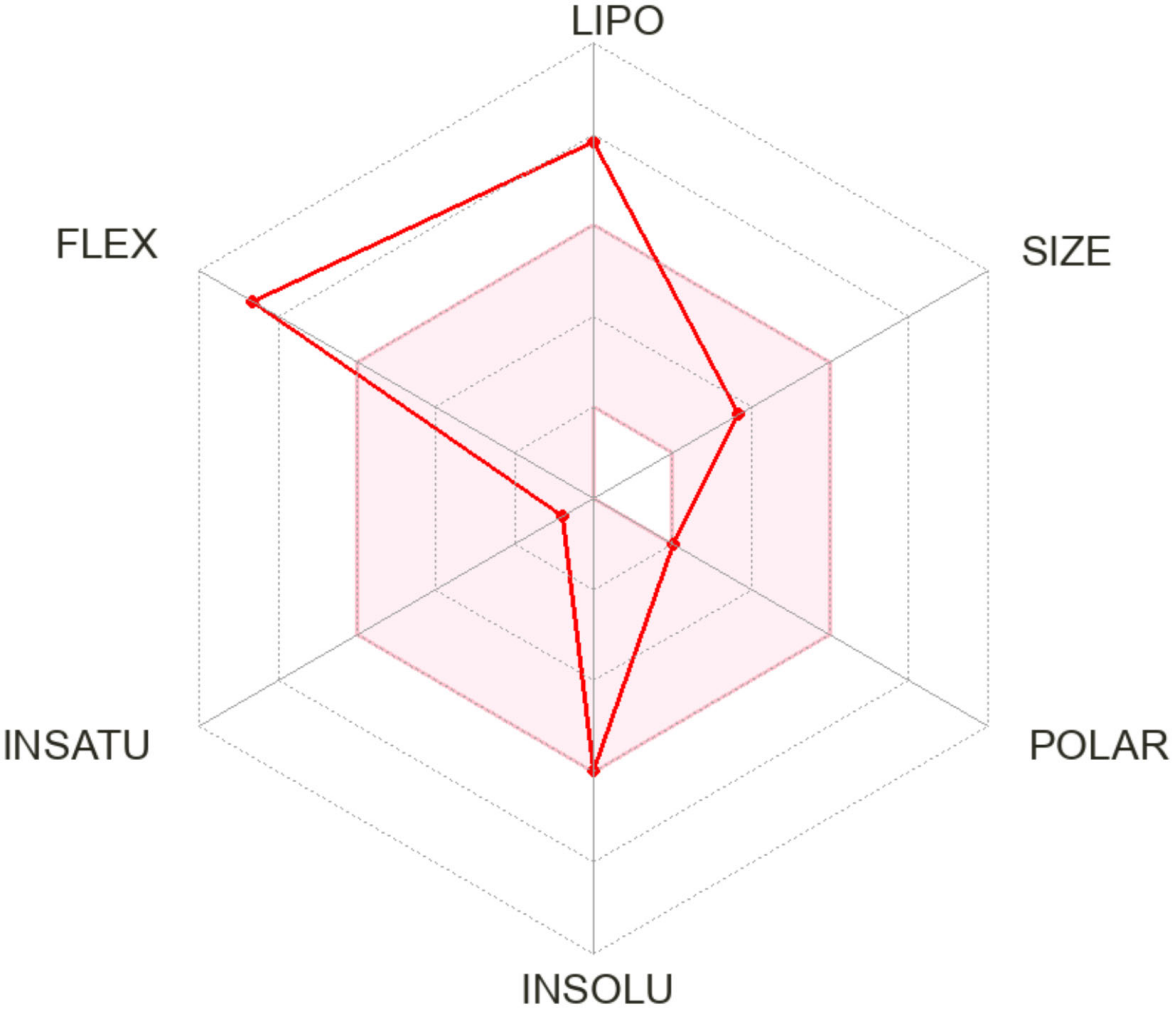
The radar chart illustrates the physiochemical properties of the phytol across six parameters: lipophilicity (LIPO), size (SIZE), polarity (POLAR), water insolubility (INSOLU), degree of unsaturation (UNSATU), and molecular flexibility (FLEX). Each axis represents a distinct property, with higher values indicating greater intensity.

The radar chart (**Figure 7**) illustrates the physiochemical attribute of phytol, emphasizing its high lipophilicity, moderate molecular size, low polarity, and significant molecular flexibility, which collectively enhance its hydrophobic interactions and adaptability to diverse molecular targets. Additional evaluations indicate moderate intestinal absorption (90.64%), low skin permeability (-2.631) and minimal blood-brain barrier penetration (0.793). As a substrate for CYP3A4 and an inhibitor of CYP1A2, phytol engages in specific metabolic pathways. Furthermore, its non-hepatotoxic, non-mutagenic and is non-carcinogenic profile reinforce its therapeutic potential while warranting further exploration for drug development (**Supplementary Tables S1**–**S3**).

### 3.10 Protein-protein interaction network analysis

**Figure 8:**
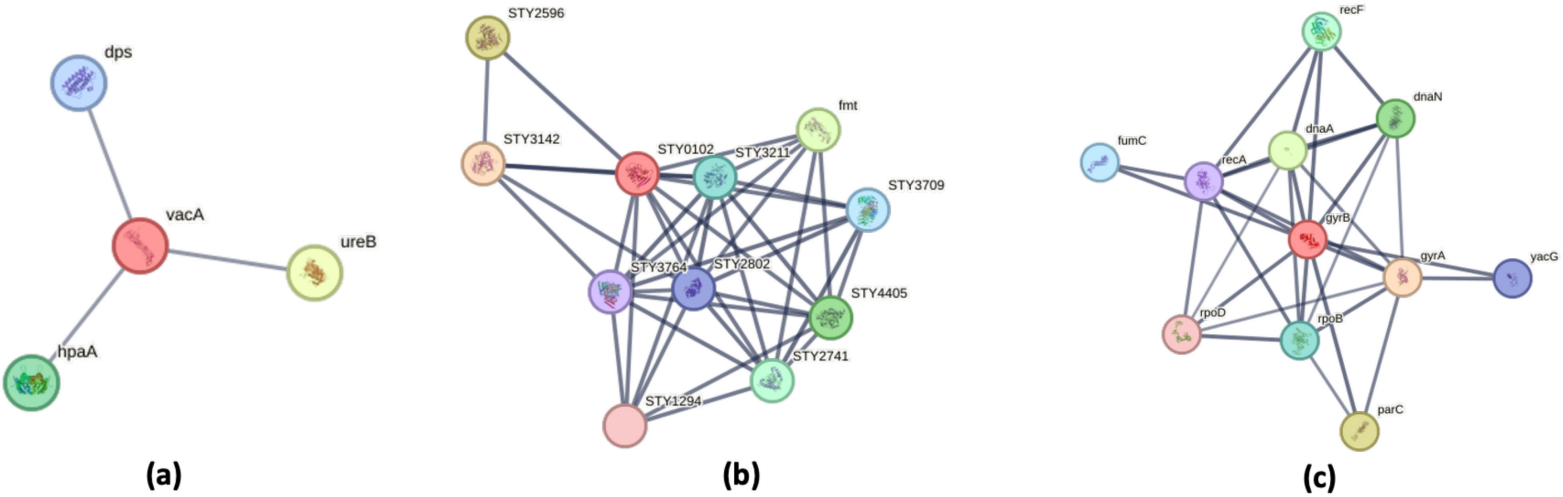
Protein–Protein Interaction Networks of Bacterial Virulence and Adaptation Factors. Protein–protein interaction (PPI) networks were generated using the STRING database, with PPI enrichment p-value: 2.04e-07. Proteins involved in localization are highlighted in red. **(a)** Helicobacter pylori: The PPI network is centered around the vacuolating cytotoxin VacA, a key virulence factor involved in host cell interaction. **(b)** Salmonella Typhi: The network centers on dihydrofolate reductase (DHFR, STY0102), a pivotal metabolic enzyme that acts as a hub connecting multiple biosynthetic pathways. **(c)** Escherichia coli: The DNA gyrase subunit B (GyrB) occupies a central position in the interaction network, underscoring its role as a master regulator of genomic processes including DNA replication, repair, and transcription.

The **Figure 8** illustrates three distinct protein-protein interaction (PPI) networks that reveal critical pathway disruptions in bacterial pathogens. In *H. pylori*, the network centers on the virulence factor VacA, which interacts with UreB (urease subunit) and HpaA (involved in nickel cation binding), demonstrating coordinated roles in host cell damage, acid neutralization. Nickel acts as a virulence determinant in *H. pylori* by serving as an essential cofactor for urease, an enzyme critical for gastric colonization through acid resistance. Notably, studies have shown that *H. pylori* accumulate nickel at concentrations 50 times higher than those found in *E. coli* (Manente et al., 2008; Shah et al., 2022). Therefore, a supply of nickel is crucial for its survival in the stomach. The interactions imply synergy—UreB may enhance VacA’s cytotoxic effects by altering local p^H^, while HpaA could localize VacA to host cell surfaces for targeted toxin delivery (Vitoriano et al., 2011). Together, these interactions likely disrupt host pathways (e.g., mitochondrial apoptosis, immune signaling) to sustain infection, offering potential targets for anti-*H. pylori* therapies (Shah et al., 2022).

In S. Typhi, dihydrofolate reductase (DHFR, STY0102) serves as a metabolic nexus (He et al., 2020), connecting folate metabolism to nucleotide synthesis through interactions with thymidylate synthase (STY3142) and folylpolyglutamate synthase (STY2596) (Joshi et al., 2022; Shen and Downs, 2024). This network not only supports bacterial proliferation but also reveals compensatory pathways that could be co-targeted with DHFR inhibitors like trimethoprim to overcome resistance.

The protein-protein interaction (PPI) network of DNA gyrase subunit B (GyrB) in *E. coli* reveals its central role as a master regulator of genomic processes (Menon and Piramanayakam, 2021). As the catalytic core of DNA gyrase, GyrB partners with GyrA to control DNA supercoiling (Nöllmann et al., 2007). The network demonstrates GyrB’s multifaceted interactions with key cellular components, it coordinates with DnaA to initiate replication and DnaN to maintain processivity (Katayama et al., 2017), interfaces with RpoB/RpoD to regulate transcription, and connects to RecF/RecA for DNA repair. Targeting GyrB alongside topoisomerase IV (ParC) could amplify the effect of quinolone antibiotics by simultaneously disrupting chromosome topology and associated pathways (Bansal and Tandon, 2011; Gurram and Azam, 2021).

Collectively, these networks expose vulnerable points in bacterial physiology—virulence factor coordination (*H. pylori*), metabolic resilience (S. Typhi), and genome-metabolism crosstalk (*E. coli*)—providing a roadmap for multitarget therapies to disrupt pathogenesis and combat antibiotic resistance.

## 4 Conclusion

Phytol demonstrated significant potential as a bioactive compound with antibacterial activities. Our findings suggest that phytol effectively inhibits bacterial growth, bactericidal effect, highlighting its potential as both an inhibitors and bactericidal agent. The variation in MIC and MBC values across different species also suggest that phytol’s antibacterial efficacy may be more pronounced against certain pathogens. The pharmacokinetic profile, as assessed through various bioinformatics tools shows a favorable molecular weight and molar refractivity, positioning it as a viable candidate for drug development. The molecular docking analysis exhibits varying binding affinities against the target proteins from different bacteria, with notable implications for its antibacterial activity. The strongest binding energy was observed with Dihydrofolate Reductase (DHFR) from *Salmonella enterica* serovar Typhi, suggesting a robust interaction that may enhance its potential as an antibacterial agent against this pathogen. These findings support the hypothesis that Phytol could serve as a promising lead compound for further investigation into its antibacterial properties, particularly against *Salmonella enterica* serovar Typhi.

## Data Availability

The original contributions presented in the study are included in the article/**Supplementary Material**. Further inquiries can be directed to the corresponding author.
