# Supplementary material for: Exploring the bioactive compounds of Carica papaya leaves: phytol’s role in combatting antibiotic-resistant bacteria
Source: Front Cell Infect Microbiol. 2025 Jul 7;15:1564787. doi: 10.3389/fcimb.2025.1564787 (PMC12277969; doi:10.3389/fcimb.2025.1564787)
Supplement: Supplementary file 1 [file Supplementaryfile1.docx]

**Supplementary material**


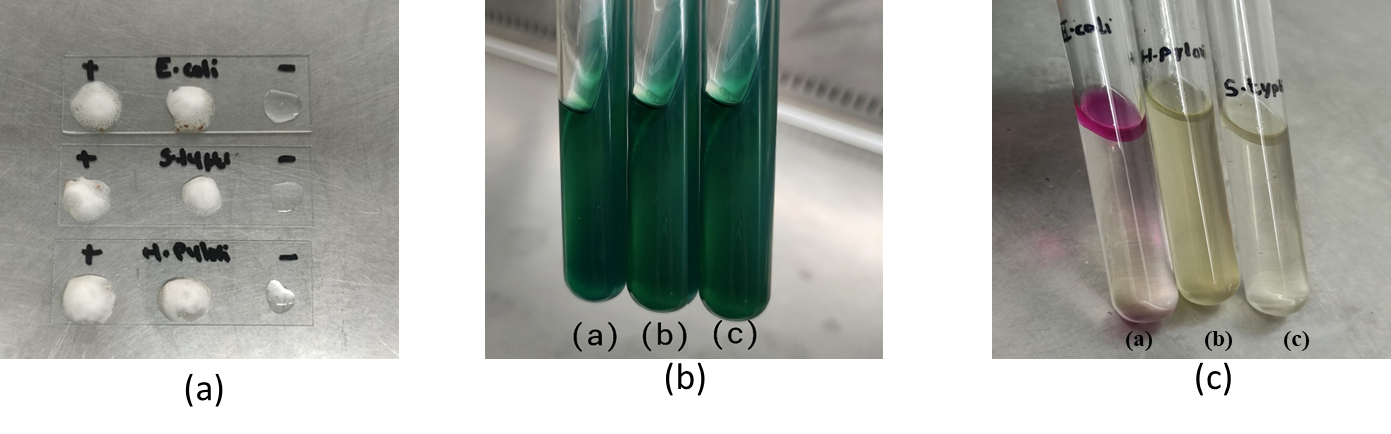


**Figure S1** (a) illustrates that *E. coli, S.* Typhi, and *H. pylori* are catalase-positive, as evidenced by bubble formation upon mixing with H₂O₂. This reaction indicates the presence of the enzyme catalase, which breaks down H₂O₂ into water and oxygen. In contrast, the negative control showed no bubble formation when mixed with H₂O₂. (b) The green color observed confirms that all three pathogens are citrate-negative, indicating their inability to utilize citrate as a sole carbon source. (c) The red/purple ring formed by *E. coli* signifies it is indole-positive, demonstrating the presence of the enzyme tryptophanase. In contrast, the yellow/green ring observed in *H. pylori* and *S.* Typhi indicates they are indole-negative and lack tryptophanase activity.


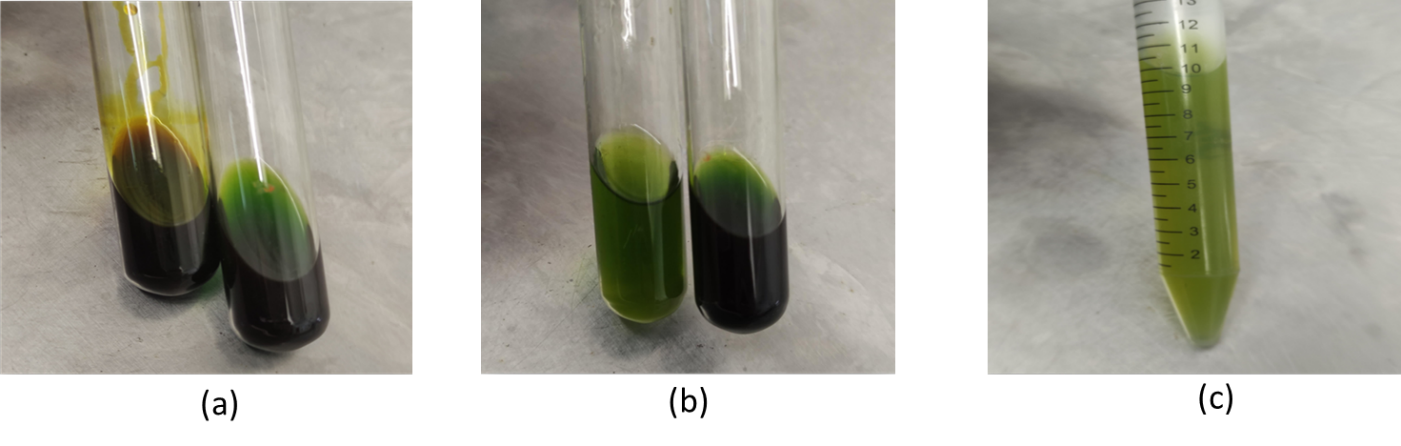


**Figure S2 |** (a) The detection of phenols was confirmed by the appearance of a brownish-black color after the addition of 3–4 drops of ferric chloride solution. (b) A noticeable color change in the extract upon the addition of a few drops of NaOH solution indicated the presence of flavonoids. (c) The absence of persistent frothing in the mixture, after adding the solvent and shaking for 15 minutes, confirmed the absence of saponins.
