# Supplementary material for: Exploring the bioactive compounds of Carica papaya leaves: phytol’s role in combatting antibiotic-resistant bacteria
Source: Front Cell Infect Microbiol. 2025 Jul 7;15:1564787. doi: 10.3389/fcimb.2025.1564787 (PMC12277969; doi:10.3389/fcimb.2025.1564787)
Supplement: Supplementary file 2 [file Table1.docx]

**Supplementary material**

**Table S1: pkCSM pharmacokinetic parameters of phytol**

| **Pharmacokinetic properties** | | **Phytol** |
| --- | --- | --- |
| **Absorption** | **Water solubility** | **-7.535** |
|  | **Caco2 permeability** | **1.399** |
|  | **Intestinal absorption (human)** | **90.643** |
|  | **Skin Permeability** | **-2.631** |
|  | **P-glycoprotein substrate** | **No** |
|  | **P-glycoprotein I inhibitor** | **No** |
|  | **P-glycoprotein II inhibitor** | **No** |
| **Distribution** | **VDss (human)** | **0.385** |
|  | **Fraction unbound (human)** | **0** |
|  | **BBB permeability** | **0.793** |
|  | **CNS permeability** | **-1.527** |
| **Metabolism** | **CYP2D6 substrate** | **No** |
|  | **CYP3A4 substrate** | **Yes** |
|  | **CYP1A2 inhibitor** | **Yes** |
|  | **CYP2C19 inhibitor** | **No** |
|  | **CYP2C9 inhibitor** | **No** |
|  | **CYP2D6 inhibitor** | **No** |
|  | **CYP3A4 inhibitor** | **No** |
| **Excretion** | **Total Clearance** | **1.686** |
|  | **Renal OCT2 substrate** | **No** |
| **Toxicity** | **AMES toxicity** | **No** |
|  | **Max. tolerated dose (human)** | **-0.301** |
|  | **hERG I inhibitor** | **No** |
|  | **hERG II inhibitor** | **Yes** |
|  | **Oral Rat Acute Toxicity (LD50)** | **1.848** |
|  | **Oral Rat Chronic Toxicity (LOAEL)** | **1.232** |
|  | **Hepatotoxicity** | **No** |
|  | **Skin Sensitization** | **Yes** |
|  | ***T.Pyriformis* toxicity** | **1.714** |
|  | **Minnow toxicity** | **-1.137** |

**Table S2: StopTox toxicity parameters of phytol**

| **Ligand** | **Acute Inhalation Toxicity**  **(%)** | **Acute Oral Toxicity**  **(%)** | **Acute Dermal Toxicity**  **(%)** | **Eye Irritation and Corrosion**  **(%)** | **Skin Sensitization**  **(%)** | **Skin Irritation and Corrosion**  **(%)** |
| --- | --- | --- | --- | --- | --- | --- |
| Phytol | 60.0 | 100 | 90 | 78 (**+**) | 60 (**+**) | 50 (**+**) |

**Table S3: Pro-Tox II toxicological parameters of phytol**

| **Classification** | **Target** | **Phytol** | |
| --- | --- | --- | --- |
|  |  | **Pre** | **Pro** |
| **Organ toxicity** | **Hepatotoxicity** | I | 0.79 |
| **Toxicity end points** | **Carcinogenicity** | I | 0.76 |
|  | **Immunotoxicity** | I | 0.99 |
|  | **Mutagenicity** | I | 0.97 |
|  | **Cytotoxicity** | I | 0.85 |
| **Tox21-Nuclear receptor signalling pathways** | **AhR** | I | 1 |
|  | **AR** | I | 1 |
|  | **AR-LBD** | I | 1 |
|  | **Aromatase** | I | 0.99 |
|  | **ER** | I | 0.99 |
|  | **ER-LBD** | I | 0.99 |
|  | **PPAR-Gamma** | I | 1 |
|  | **nrf2/ARE** | I | 0.98 |
| **Tox21-Stress response pathways** | **HSE** | I | 0.98 |
|  | **MMP** | I | 0.99 |
|  | **p53** | I | 1 |
|  | **ATAD5** | I | 1 |
